# Glycolysis-related gene signatures in spinal cord injury pathophysiology identification through integrative gene expression analysis

**DOI:** 10.3389/fgene.2026.1759563

**Published:** 2026-02-16

**Authors:** Xiaoqin Liu, Zhuang Wang, Jiating Hu, Guodong Shi, Xin Ai

**Affiliations:** 1 Yan’an Medical College of Yan’an University, Yan’an, China; 2 Department of Radiology, The Affiliated Hospital of Yan’an University, Yan’an, China; 3 Department of Neurosurgery, The Affiliated Hospital of Yan’an University, Yan’an, China

**Keywords:** glycolysis, immune microenvironment, machine learning, single cell, spinal cord injury

## Abstract

**Background:**

Spinal cord injury (SCI) is a debilitating condition that significantly impacts patients’ mobility and quality of life, posing a substantial economic burden on healthcare systems. Increasing evidence suggests that metabolic reprogramming, particularly glycolysis, is involved in inflammatory responses following SCI. This study aims to systematically investigate the association between the role of glycolysis-related genes (GRGs) and SCI, and to identify potential candidate biomarkers.

**Methods:**

The GSE151371 dataset was retrieved from the Gene Expression Omnibus (GEO) to identify differentially expressed genes (DEGs), which were subsequently subjected to Weighted Gene Co-expression Network Analysis (WGCNA) to pinpoint glycolysis-associated modules. Hub genes associated with SCI were initially identified through machine learning algorithms and subsequently evaluated using the independent GSE45006 dataset. Immune infiltration in SCI was profiled by single-sample gene set enrichment analysis (ssGSEA) and correlated with hub gene expression. After establishing TF-miRNA-mRNA and protein-chemical networks, hub gene expression patterns were characterized by scRNA-seq and further validated experimentally *in vivo* by qRT-PCR and Western blotting.

**Results:**

From 1,138 DEGs, WGCNA identified 704 in glycolysis-associated modules. Intersecting these with GRGs yielded 13 candidates. Subsequent machine learning pinpointed six hub genes (*ALK*, *GGH*, *IRS1*, *PPARG*, *SLC1A3*, *UBTD1*), of which only *IRS1* and *UBTD1* showed consistent expression patterns in an external dataset. ssGSEA identified 20 differentially abundant immune cell types in SCI. Subsequently, *IRS1* expression was associated with activated T cells and natural killer (NK) cells, while *UBTD1* expression correlated with activated dendritic cells, monocytes, and neutrophils. scRNA-seq revealed that *Irs1* was mainly expressed in endothelial and epithelial cells, while *Ubtd1* was broadly expressed, with higher levels in endothelial cells and microglia. qRT-PCR revealed significant upregulation of *Ubtd1* in the SCI group, whereas *Irs1* expression did not differ significantly. Western blot further confirmed elevated UBTD1 protein levels in SCI compared with the Sham group.

**Conclusion:**

Our integrative transcriptomic and experimental analyses suggest that *UBTD1*, a glycolysis-related gene, as significantly associated with SCI and immune cell infiltration, highlighting its potential as a biomarker and suggesting its role in metabolic–immune interactions post-SCI.

## 1 Introduction

Spinal cord injury (SCI) represents a major public health challenge, as it results in significant lifelong disabilities and imposes a considerable economic burden on healthcare systems and society (Crispo et al., 2023). The intricate nature of SCI, characterized by a cascade of biological events following initial trauma, necessitates a comprehensive understanding of its underlying mechanisms to develop effective therapeutic strategies (Ahuja et al., 2017; Zheng and Tuszynski, 2023). Despite advancements in medical interventions, including surgical options and rehabilitation therapies, the restoration of function and improvement in the quality of life for affected individuals remain limited (Hu et al., 2023). Therefore, there is an urgent need for innovative research that delves into the molecular alterations that accompany SCI and their potential therapeutic implications. Glycolysis, a fundamental metabolic pathway for energy production in cells, has increasingly been recognized as a key modulator of neuroinflammatory responses mediated by microglia (Hua et al., 2024; Yu et al., 2023). In response to pathological stimuli, such as SCI and neurodegenerative diseases, microglia undergo extensive shifts in their metabolic profile (Sabogal-Guáqueta et al., 2023). Previous studies have identified a clear association between the M1 polarization of microglia and an upregulation of glycolytic activity in the context of SCI (Ding et al., 2025). After the onset of primary SCI, the affected microenvironment rapidly experiences ischemia and hypoxia, which triggers a metabolic switch in microglia, infiltrating macrophages, and astrocytes, from oxidative phosphorylation to glycolysis (Yu et al., 2023; Orihuela et al., 2016; Viola et al., 2019). Although this metabolic shift initially supports the increased energy requirements of activated immune cells, prolonged activation of this pathway amplifies the inflammatory response via several mechanisms. This implies that targeting metabolic reprogramming may offer a promising approach for therapeutic intervention.

**FIGURE 1:**
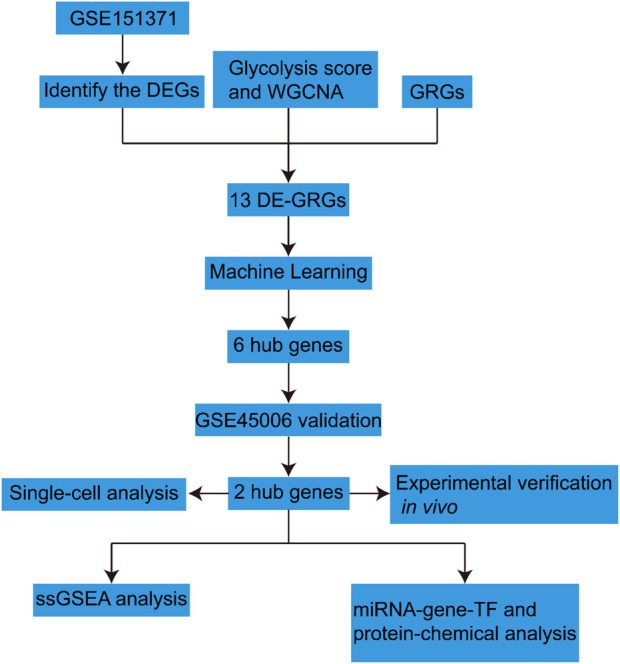
Overview of the study workflow.

To address these gaps in knowledge, our study employs a multifaceted methodological approach that integrates large-scale gene expression datasets, advanced analytical techniques such as weighted gene co-expression network analysis (WGCNA) and machine learning algorithms. This comprehensive strategy is designed to elucidate the intricate relationships between glycolysis-related genes (GRGs) and SCI, thereby identifying potential biomarkers and therapeutic targets. The primary objective of this research is to elucidate the specific roles of GRGs in the context of SCI and to assess their potential as therapeutic targets. By analyzing the SCI-related datasets from GEO and employing rat models, we examined the expression patterns of GRGs and their contribution to risk evaluation in the context of SCI progression, with a particular emphasis on the modulation of immune microenvironments (Figure 1).

## 2 Materials and methods

### 2.1 Data acquisition

Datasets GSE151371, GSE45006, and GSE162610 were sourced from the GEO (Gene Expression Omnibus) database (https://www.ncbi.nlm.nih.gov/geo/) (Edgar et al., 2002). The GSE151371 dataset includes gene expression profiles of peripheral white blood cells (WBCs) along with clinical data from 38 SCI patients and 10 healthy individuals (Kyritsis et al., 2021). Probes were mapped to their respective gene symbols using the annotation information from the GPL20301 [Illumina HiSeq 4,000 (*Homo sapiens*)] platform. The GSE45006 microarray expression dataset for SCI rats was generated using the GPL1355 [Rat230_2] Affymetrix Rat Genome 230 2.0 Array platform. In this dataset, SCI was induced in rats using an aneurysm clip impact-compression injury model on the thoracic spinal cord (T7). This dataset includes the Sham group (n = 4) and the SCI group, with samples collected at 1, 3, and 7 days post-injury (n = 4 per time point). Although GSE151371 and GSE45006 differ in species and tissue origin, GSE45006 was incorporated as a supportive external validation dataset to assess the consistency of glycolysis-related gene expression patterns at the transcriptomic and pathway levels, rather than to establish direct tissue or species equivalence. The scRNA-seq dataset GSE162610 includes data from normal and injured spinal cords at 1, 3, and 7 days post-injury of wild-type mice. This dataset was used to characterize the cellular distribution of candidate hub genes within the spinal cord microenvironment, thereby providing complementary cell-type–specific context for the transcriptomic findings derived from bulk RNA-seq analysis.

The GeneCards database (https://www.genecards.org/) provides extensive gene information for humans (Stelzer et al., 2016). By searching with the keyword “glycolysis”, we selected genes classified as ‘Protein Coding’ and with a relevance score exceeding 1.00, thereby identifying glycolysis-related genes (GRGs). This search resulted in the identification of 1,572 GRGs, which are listed in Supplementary Table S1.

### 2.2 PCA-based calculation of glycolysis score

To quantify glycolytic transcriptional activity in each sample, a predefined set of glycolysis-related genes was extracted from the transcriptomic data, and principal component analysis (PCA) was performed using the expression matrix of these genes. PCA identifies orthogonal linear combinations of genes that successively maximize variance within the selected gene set. In this study, the first two principal components (PC1 and PC2) captured the dominant axes of coordinated transcriptional variation among glycolysis-related genes. As PC1 and PC2 together explained the majority of the variance within this gene set, a composite glycolysis score was constructed by summing the PC1 and PC2 scores for each sample. This PCA-based score was used as an empirical measure to summarize overall glycolytic transcriptional activity, consistent with previous studies (Sun et al., 2021). This score summarizes coordinated transcriptional variation within GRGs.

### 2.3 Weighted gene Co-expression network analysis (WGCNA)

In this study, WGCNA was used to identify gene modules strongly associated with glycolysis, utilizing the “WGCNA” package (version 1.73) in R software (Langfelder and Horvath, 2008). Hierarchical clustering was applied to the study samples to identify and exclude outliers. A soft-thresholding power of β = 12 was chosen to build a scale-free network and compute the topological overlap matrix. The correlation between each module and the glycolysis score was evaluated. Modules with a high correlation coefficient were identified as key modules strongly associated with glycolysis. A correlation coefficient exceeding 0.5 and a significance level of *P* < 0.05 were set as the criteria for module-phenotype correlation. Genes from the key module were compared with glycolysis-related genes and DEGs, leading to the identification of a set of glycolysis-associated DEGs (GR-DEGs).

### 2.4 Screening of DEG

The raw GEO data was normalized using the ‘NormalizeBetweenArray’ function in R. Differentially expressed genes (DEGs) between the HC and SCI groups in the GSE151371 dataset were identified using the “Limma” package, which was found to be the most efficient tool. A log fold change (LogFC) threshold of >0.5 and an adjusted *P*-value <0.05 were applied to determine gene differential expression.

### 2.5 Identification of feature genes using machine learning algorithms

In this study, we utilized the Bagged Trees, Bayesian Networks, Support Vector Machine (SVM), Random Forest, XGBoost, Boruta, Learning Vector Quantization (LVQ), and 1,000 iterations of 10-fold cross-validation with Least Absolute Shrinkage and Selection Operator (LASSO) algorithms to pinpoint the key genes linked to glycolysis. Genes that were consistently recognized as feature genes by at least 7 different algorithms, based on their classification efficacy, were classified as key hub genes involved in the interaction between SCI and glycolysis. The interactions between these crucial intersecting genes were subsequently visualized using the “Upset” application from the R package. It should be noted that these machine learning algorithms were used for feature selection and robustness assessment rather than for constructing a single optimized predictive classifier.

### 2.6 Pathway analysis of the two hub genes

To investigate the regulatory pathways and biological functions linked to the two hub genes, single-gene GSEA analysis was conducted. A significance threshold of adjusted *P*-value <0.05 was applied. The top five enriched pathways for both upregulated and downregulated genes were displayed separately, based on their enrichment scores.

### 2.7 TF–miRNA regulatory interaction network

Transcription factors (TFs) are proteins that interact with DNA at specific sequences to control the process of transcription. Through their ability to recognize unique DNA motifs, TFs influence chromatin configuration and transcriptional activity, orchestrating the regulation of gene expression across the genome (Lambert et al., 2018). MicroRNAs (miRNAs) are a class of short, endogenous non-coding RNAs that regulate gene expression by either degrading target mRNAs or preventing their translation (Bartel, 2004). To elucidate the dysregulation of gene expression in diverse physiological and disease contexts, it is essential to explore the intricate transcriptional regulatory networks that govern the interactions between TFs and miRNAs (Bartel, 2004; Zaborowski and Walther, 2020; Iwama et al., 2011). NetworkAnalyst (http://www.networkanalyst.ca) is an online tool designed for advanced meta-analysis of gene expression data (Xia et al., 2015). *IRS1* and *UBTD1* were analyzed using NetworkAnalyst to construct a TF–miRNA coregulatory network. Regulatory interaction data curated from the literature were gathered from RegNetwork (http://www.regnetworkweb.org/) (Liu et al., 2015). The relevant results were visualized using Cytoscape.

### 2.8 Interactions between proteins and chemicals

Using the NetworkAnalyst platform, compounds that interact with the hub genes were retrieved from The Comparative Toxicogenomics Database (CTD), and a protein-chemical interaction network was then built based on these interactions. Cytoscape was employed to visualize the relevant results.

### 2.9 Single-cell RNA sequencing data processing and analysis

The analysis of single-cell RNA sequencing data was mainly carried out using the “Seurat (v 5.1.0)” package (Hao et al., 2021). To maintain data quality, cells with fewer than 200 detected genes or more than 20% mitochondrial gene content were excluded. Batch effects were corrected using the “Harmony” package (v.1.2.1) (Korsunsky et al., 2019). Data normalization was performed using the ‘lognormalization’ function, and highly variable genes were selected with the ‘FindVariableFeatures’ function. We applied the tSNE algorithm to classify cell types, generating a unified map that reveals structural patterns across various scales, making it especially valuable for high-dimensional data. Clustering was performed with the Louvain algorithm, and marker genes specific to each cell subpopulation were identified through the ‘FindAllMarkers’ function. Cell type annotation was initially carried out automatically using the “SingleR” package.

### 2.10 Animal model

The animals used in this study were sourced from the Laboratory Animal Center at Xi’an Jiaotong University (Animal License No. SCXC [Shan] 2023–002). All experimental protocols were approved by the Animal Ethics Committee of Yan’an University and carried out in strict compliance with both institutional and national regulations governing the ethical treatment and use of animals. A total of 12 female Sprague Dawley (SD) rats (8 weeks old) were randomly assigned to two groups: the Sham group and the SCI group. In the Sham group, a laminectomy was performed without inducing spinal cord injury. The SCI model was created following a modified version of the Allen method, as outlined in the existing literature (Liu et al., 2021). Briefly, the animals were anesthetized by intraperitoneal injection of 1% pentobarbital sodium (40 mg/kg, intraperitoneal injection), and then positioned on a surgical table. The T10 vertebra was exposed by removing the lamina using bone cutting forceps. To induce the injury, a 10 g weight was dropped from a height of 3 cm directly onto the exposed spinal cord. To prevent infection, penicillin (Dingjian, Sichuan, China) was administered subcutaneously for three consecutive days at a daily dose of 2.5 mg/kg. Bladder voiding was manually facilitated twice daily through the application of abdominal pressure until the animals were able to urinate spontaneously. Seventy-two hours following SCI induction, the SD rats were anesthetized through an overdose of 1% pentobarbital sodium administered via intraperitoneal injection. A segment of spinal cord tissue, 1.5 cm in length from the center of the injury site, was carefully excised for further analysis.

### 2.11 RNA extraction and qRT-PCR analysis

Total RNA was isolated from spinal tissues using the TRIzol Total RNA Purification Kit (5003050, Simgen, China), and cDNA synthesis was carried out with the NovoScript® Plus All-in-one 1st Strand cDNA Synthesis SuperMix (E047-01A, Novoprotein, China). Target gene expression was quantified using the NovoStart® SYBR qPCR SuperMix Plus (E096-01A, Novoprotein, China). The relative expression levels were normalized to β-actin and calculated using the 2^−ΔΔCt^ method. The primer sequences are presented in Table 1.

**TABLE 1:** Primers used in quantitative real-time PCR analysis.

Gene	Sequence (5′–3′)
*Irs1*	Forward: CTG​CAT​AAT​CGG​GCA​AAG​GC
​	Reverse: GCC​CGT​GTC​ATA​GCT​CAA​GT
*Ubtd1*	Forward: GTT​GGG​TAA​GTC​AGG​TCG​CA
​	Reverse: AGG​GCA​TCC​CAG​ATC​TCC​TT
β-actin	Forward: CCC​ATC​TAT​GAG​GGT​TAC​GC
​	Reverse: TTT​AAT​GTC​ACG​CAC​GAT​TTC

### 2.12 Western blotting

Proteins were extracted from spinal tissues, and their concentrations were quantified using the BCA method. The extracted proteins were separated by SDS-PAGE and subsequently transferred to a PVDF membrane (Merck, Germany). The membrane was blocked with 5% skimmed milk for 1 h to prevent non-specific binding. Primary antibodies were applied to the membrane overnight, including rabbit anti-UBTD1 (1:1,000 dilution, DF15663, Affinity) and rabbit anti-β-Tubulin (1:4,000 dilution, YM8332, ImmunoWay). Following washing steps, the membrane was incubated with HRP-conjugated goat anti-rabbit IgG (H + L) (1:10,000 dilution, GB111738-100, Servicebio) for 2 h at room temperature. After additional washing, the protein bands were visualized using ECL reagent, and the results were captured and documented using a chemiluminescence imaging system.

### 2.13 Statistical analysis

All analyses were conducted using R version 4.2.1. Data are presented as means ± standard error of the mean (SEM) from at least three independent experiments and were analyzed using GraphPad Prism 9.3.1. The Wilcoxon rank-sum test and Welch’s t-test were employed to compare the expression differences between unpaired samples from the two groups. *P*-values were interpreted according to the following scale of significance: asterisks denote increasing levels of significance (*P < 0.05, **P < 0.01, ***P < 0.001), while ‘ns’ denotes non-significance.

## 3 Results

### 3.1 Construction of weighted gene Co-expression network

**FIGURE 2:**
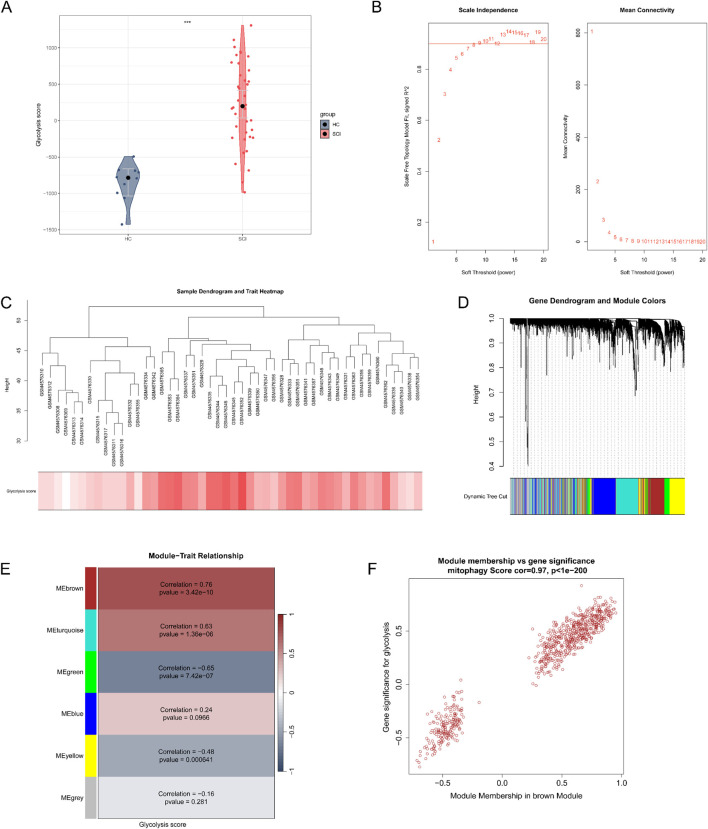
Identification of glycolysis-related modules through WGCNA. **(A)** Glycolysis scores between SCI and HC samples. **(B)** The scale-free topology model fit index as a function of the soft-thresholding power was plotted. A soft-thresholding power of 12 was selected to maintain a scale-free topology in the network construction. **(C)** Clustering was conducted using the transcriptome data from the GSE151371 dataset, where color intensity indicates the glycolysis scores. **(D)** Coexpression modules were identified through hierarchical clustering, with unique colors assigned to each module. In the resulting dendrogram, each branch corresponds to a gene, and genes that cluster together are assigned the same module color. **(E)** A heatmap of module-trait associations showing the link between modules and glycolysis scores. Positive correlations are depicted in red, whereas negative correlations are shown in blue. **(F)** Correlation analysis was performed between module membership (MM) in the brown module and gene significance (GS) for glycolysis.

PCA was performed based on the expression of GRGs to calculate the glycolysis score for each sample. The glycolysis score was significantly higher in SCI samples compared to HC samples (Figure 2A). Using the GSE151371 dataset from GEO, clustering analysis was carried out, and a soft-thresholding power of β = 12 was chosen, ensuring an *R* ^2^ value greater than 0.9 and a relatively high mean connectivity (Figure 2B). Under the parameter settings of minModuleSize = 100 and mergeCutHeight = 0.25, six modules were identified (Figures 2C,D). The MEbrown module demonstrated the strongest correlation with the score, exhibiting a correlation coefficient of r = 0.76 and a *P*-value of 3.42e-10, as shown in Figure 2E. Moreover, the GS and MM values in the brown module were strongly correlated, suggesting that this module exhibited the most significant association with glycolysis (Figure 2F). Based on this, we selected the MEbrown module, which consists of 704 genes, as the key module for further analysis.

### 3.2 Comparative analysis of GRG expression in SCI and HC samples

**FIGURE 3:**
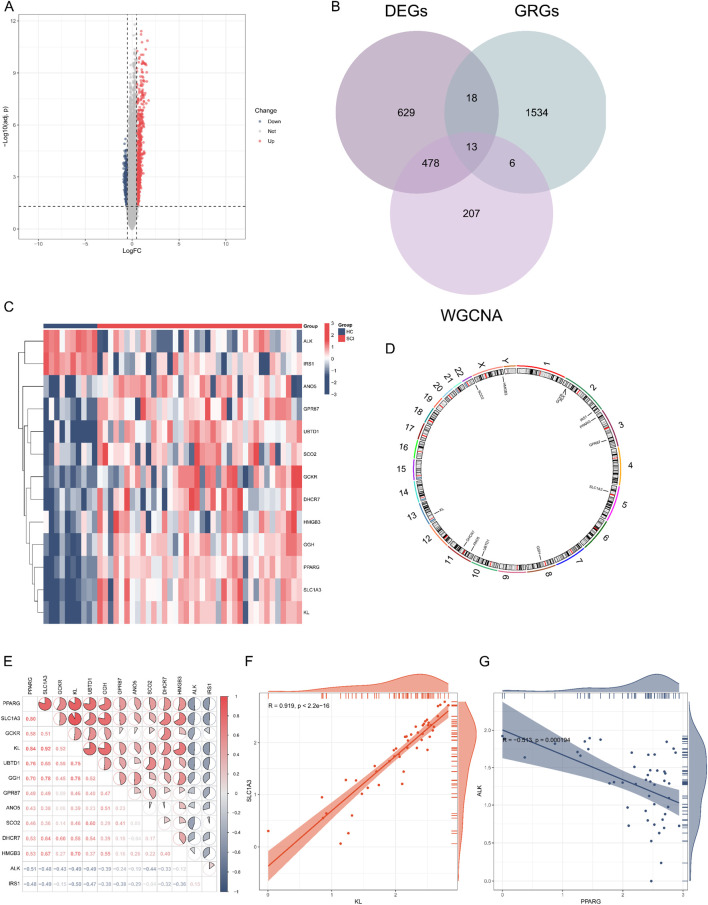
Expression profiles of GRGs in SCI and HC samples. **(A)** A volcano plot illustrating the DEGs in the GSE151371 dataset, selected based on the thresholds |log2FC| > 0.5 and adjusted *P*-value <0.05, comparing SCI patients to HC. **(B)** A Venn diagram was employed to identify DEGs associated with glycolysis. **(C)** Heatmap showing the expression of GR-DEGs between SCI and HC. **(D)** Chromosomal locations of the 13 GR-DEGs. **(E)** Spearman correlation analysis of the 13 GR-DEGs in SCI samples. **(F,G)** Scatterplots were used to illustrate the strongest correlations among the 13 GR-DEGs: KL and SLC1A3 exhibited a positive correlation, while PPARG and ALK showed a negative correlation.

Differential expression analysis of the GSE151371 dataset identified a total of 1,138 DEGs, with 707 genes being upregulated and 431 genes downregulated, as depicted in the volcano plot (Figure 3A). By intersecting the key WGCNA module genes, DEGs, and GRGs, a total of 13 GR-DEGs were identified (Figure 3B). Additionally, a heatmap was generated to illustrate the expression patterns of the 13 GR-DEGs. The results showed that *ALK* and *IRS1* were downregulated, while *PPARG*, *SLC1A3*, *GCKR*, *KL*, *UBTD1*, *GGH*, *GPR87*, *ANO5*, *SCO2*, *DHCR7*, and *HMGB3* were upregulated in SCI (Figure 3C). The positions of the 13 GR-DEGs on the chromosomes were mapped using the “RCircos” package for visualization (Figure 3D) (Zhang et al., 2013). The correlation analysis of the 13 GR-DEGs revealed significant interconnections among these genes, indicating a strong mutual correlation. As shown in Figures 3E–G, the correlation heatmap illustrates the overall relationships among the 13 GR-DEGs, while Figures 3F,G display the strongest positive and negative correlations, respectively.

### 3.3 Key genes were identified through the application of machine learning

**FIGURE 4:**
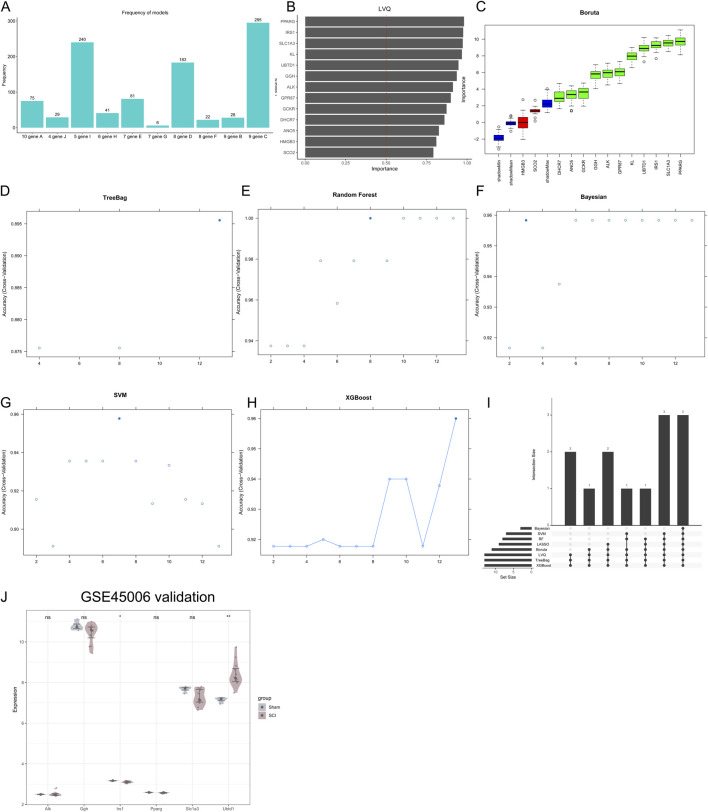
Machine learning approaches were employed to investigate the hub genes involved in the interactions between SCI and glycolysis. The outcomes from the application of various machine learning algorithms to the GSE151371 dataset are illustrated as follows: LASSO **(A)**, LVQ **(B)**, Boruta **(C)**, Bagged Tree **(D)**, Random Forest **(E)**, Bayesian **(F)**, SVM **(G)**, and XGBoost **(H)**. Panel **(I)** provides a summary of the hub genes identified as pivotal for the interactions between SCI and glycolysis by all eight machine learning algorithms. **(J)** The expression of Irs1 and Ubtd1 were validated in the dataset GSE45006. Data are presented as mean ± SEM. *P < 0.05, **P < 0.01, ***P < 0.001; ns, not significant.

The GSE151371 dataset was used to apply machine learning, which facilitated the identification of 13 GR-DEGs associated with the SCI. The intersection of findings from various machine learning algorithms, including Lasso-Logistic regression with 1,000 iterations (Figure 4A), the LVQ algorithm (Figure 4B), Boruta (Figure 4C), Bagged Trees (Figure 4D), Random Forest (Figure 4E), Bayesian Networks (Figure 4F), SVM (Figure 4G), and XGBoost (Figure 4H), revealed six genes that are significantly associated with the interaction between SCI and glycolysis (Figure 4I). These genes are *ALK*, *GGH*, *IRS1*, *PPARG*, *SLC1A3*, and *UBTD1*. To validate the six hub genes, the GSE45006 dataset was utilized, and the analysis demonstrated that only *Irs1* and *Ubtd1* exhibited significant differential expression between the SCI and Sham groups (Figure 4J).

### 3.4 Immune cell infiltration

**FIGURE 5:**
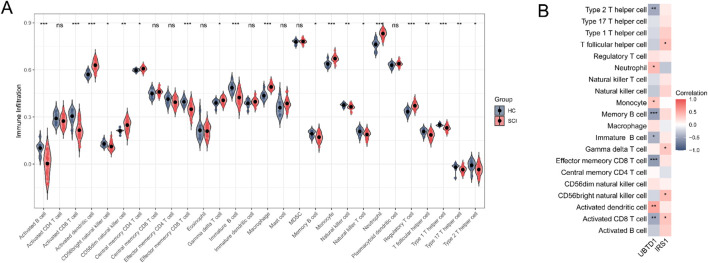
Analysis of immune infiltration levels associated with the two hub genes. **(A)** ssGSEA enrichment analysis to assess the differential expression of immune cells between SCI and HC samples. **(B)** The correlation between the two hub genes and immune cells in SCI. Data are presented as mean ± SEM. *P < 0.05, **P < 0.01, ***P < 0.001; ns, not significant.

The relative abundance of 28 immune cell types was quantitatively evaluated using the ssGSEA method, and comparisons were conducted between the SCI and HC groups based on the peripheral blood transcriptomic data. Significant disparities in the abundance of 20 immune cell types between the two groups were identified through the Wilcoxon rank-sum test. Specifically, the SCI group showed significantly higher infiltration levels of activated dendritic cells, CD56dim natural killer cells, central memory CD4 T cells, gamma delta T cells, macrophages, monocytes, neutrophils, and regulatory T cells. In contrast, the infiltration levels of activated B cells, activated CD8 T cells, CD56bright natural killer cells, effector memory CD8 T cells, immature B cells, memory B cells, natural killer cells, natural killer T cells, T follicular helper cells, type 1 T helper cells, type 17 T helper cells, and type 2 T helper cells were significantly lower (Figure 5A). Further correlation analysis revealed a significant positive correlation between *IRS1* and the abundance of activated CD8 T cells, CD56bright natural killer cells, gamma delta T cells, and T follicular helper cells in SCI (Figure 5B). Additionally, *UBTD1* showed a significant positive correlation with activated dendritic cells, monocytes, and neutrophils, while demonstrating a negative correlation with activated CD8 T cells, effector memory CD8 T cells, immature B cells, memory B cells, and type 2 T helper cells in SCI (Figure 5B). These findings reveal distinct alterations in immune cell populations within systemic circulation of SCI patients, suggesting a potential link between systemic immune responses and the identified hub genes.

### 3.5 Analysis of pathways associated with the two hub genes

**FIGURE 6:**
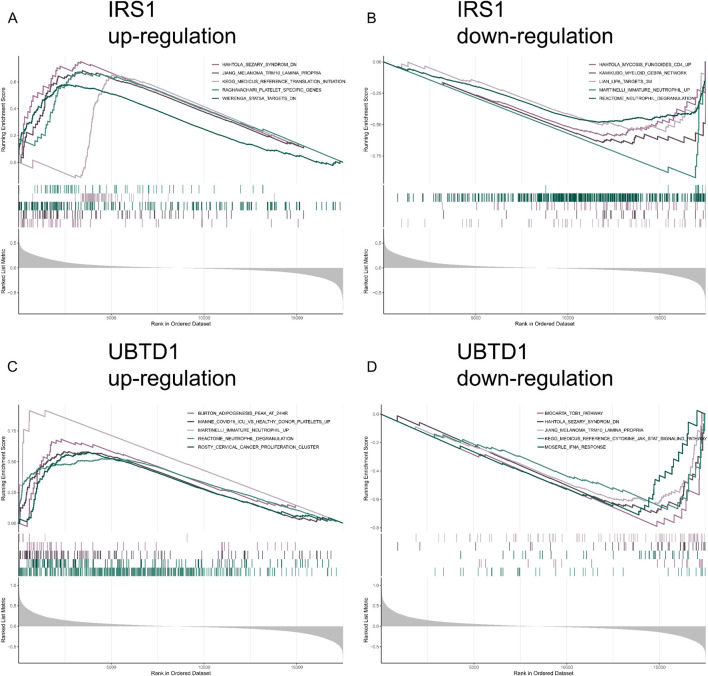
Pathway and functional analysis of the two hub genes. **(A,B)** The top five pathways positively correlated with *IRS1* and *UBTD1*. **(C,D)** The top five pathways negatively correlated with *IRS1* and *UBTD1*.

A single-gene GSEA analysis was performed to explore the biological functions and associated pathways of the two candidate hub genes. The upregulation of *IRS1* is linked to immune-related pathways, platelet-specific genes, and translation initiation processes (Figure 6A). The downregulation of *IRS1* is associated with immune functions, neutrophil activity, and the regulation of myeloid cells (Figure 6B). These findings underscore the diverse biological functions of *IRS1* in regulating both immune and cellular activities. The upregulation of *UBTD1* is associated with immune response regulation, neutrophil activity, and cancer cell proliferation, particularly in the context of infection and cancer (Figure 6C). The downregulation of *UBTD1* is linked to immune response mechanisms, particularly cytokine signaling and interferon pathways, while also being linked to diseases like Sezary syndrome and melanoma (Figure 6D). These results emphasize the broad and complex roles of *IRS1* and *UBTD1* in regulating immune and cellular activities across different biological contexts.

### 3.6 TF-gene-miRNA interactions networks

**FIGURE 7:**
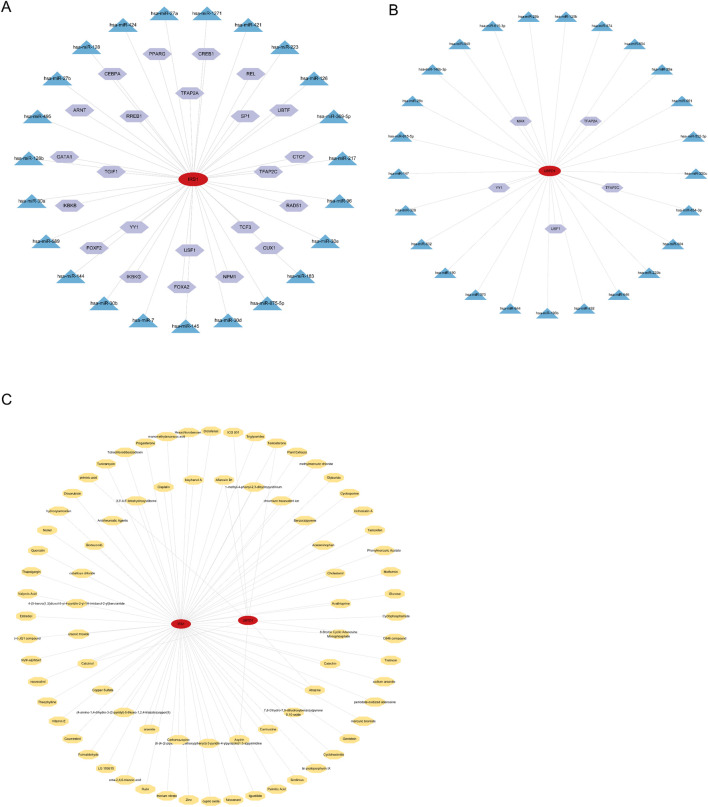
Development of the regulatory network for hub genes. Integrated TF–miRNA interaction networks for *IRS1* **(A)** and *UBTD1* **(B)** are presented. In the diagram, hexagons represent TF, triangles denote miRNA, and ellipses represent mRNAs. **(C)** The protein-chemical interaction network is depicted, where octagon nodes represent chemicals, and proteins that interact with these chemicals are shown as elliptical nodes.

The TF–miRNA coregulatory network for *IRS1* is composed of 48 nodes and 47 edges. The analysis indicates that *IRS1* is modulated by 23 TFs and 23 miRNAs (Figure 7A). The TF–miRNA coregulatory network for *UBTD1* consists of 31 nodes and 30 edges. The findings demonstrate that *UBTD1* is influenced by 5 TFs and 25 miRNAs (Figure 7B).

### 3.7 Protein-chemical interaction networks

Protein-chemical interaction networks are crucial for understanding the mechanisms underlying disease development and can aid in the drug discovery process. In this study, protein-chemical interaction networks for the candidate hub genes were constructed, consisting of 81 nodes and 84 edges. The compounds that interact with both IRS1 and UBTD1 include Aflatoxin B1, Atrazine, Testosterone, Aspirin, and Calcitriol (Figure 7C).

### 3.8 Results of single-cell analysis

**FIGURE 8:**
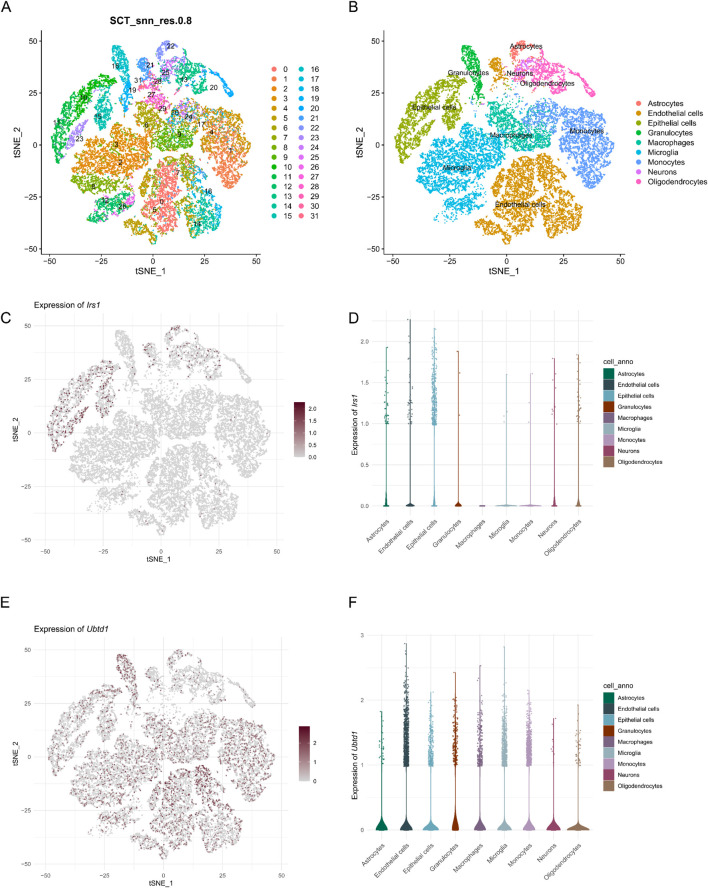
Single-cell transcriptomic analysis of spinal cord tissue. **(A)** t-SNE-based visualization depicting the distribution of 31 unique cell clusters. **(B)** t-SNE visualization of various cell types including astrocytes, endothelial cells, epithelial cells, granulocytes, macrophages, microglia, monocytes, neurons, and oligodendrocytes. **(C)** t-SNE visualization illustrating the expression pattern of *Irs1*. **(D)** The violin plot illustrates the distribution of *Irs1* expression levels in various cell types. **(E)** t-SNE visualization illustrating the expression pattern of *Ubtd1*. **(F)** The violin plot illustrates the distribution of *Ubtd1* expression levels in various cell types.

To explore the potential involvement of *IRS1* and *UBTD1* in SCI at the single-cell resolution, we performed an analysis of the scRNA-seq data from the GSE162610 dataset. A total of 28,936 cells derived from C57BL/6 mouse spinal cord samples were processed, followed by dimensionality reduction, clustering, and t-SNE visualization of the dataset. Through analysis, 31 distinct cell clusters were characterized, and marker genes were identified using the “FindAllMarkers” package (Figure 8A). Using these marker genes, a total of 162,610 cells were classified into nine distinct cell types: astrocytes, endothelial cells, epithelial cells, granulocytes, macrophages, microglia, monocytes, neurons, and oligodendrocytes (Figure 8B). *Irs1* was predominantly expressed in endothelial and epithelial cells (Figures 8C,D). We found *Ubtd1* expression across multiple cell types, with elevated levels found in endothelial cells and microglia (Figures 8E,F).

### 3.9 Experimental evaluation of candidate hub genes in the SCI animal model

**FIGURE 9:**
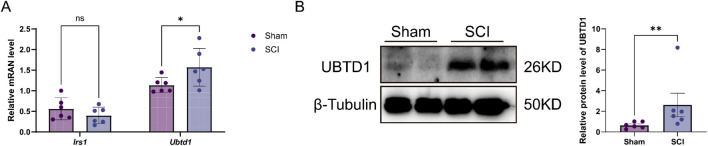
mRNA and protein expression analysis of the two hub genes in the Sham and SCI rat models. **(A)** qRT-PCR analysis of *Irs1* and *Ubtd1* mRNA expression levels in the spinal tissue from the SCI group compared with the Sham group. **(B)** Representative Western blot bands showing UBTD1 protein expression in the Sham and SCI groups. Data are presented as mean ± SEM. *P < 0.05, **P < 0.01, ***P < 0.001; ns, not significant.

To experimentally validate the roles of *IRS1* and *UBTD1* in SCI, we conducted RT-qPCR and Western blot analyses to assess the mRNA and protein levels of *IRS1* and *UBTD1* in spinal tissue from the SCI group compared to the Sham group. qPCR validation revealed a significant upregulation of *Ubtd1* in the SCI group versus the Sham group, consistent with the bioinformatic predictions. In contrast, *Irs1* mRNA levels did not exhibit a statistically significant difference (Figure 9A). Furthermore, the Western blot analysis demonstrated a notable increase in UBTD1 expression in the spinal tissue of the SCI group compared to the Sham group (Figure 9B), confirming its robust dysregulation at both transcriptional and translational levels. Consequently, based on the cross-validation results, UBTD1 was identified as the primary hub gene for further discussion.

## 4 Discussion

SCI is a grievous condition that significantly diminishes the mobility and quality of life for affected individuals (Anjum et al., 2020). The economic ramifications of SCI are profound, burdening healthcare systems due to the extensive rehabilitation and long-term care demands. Despite significant research aimed at understanding the intricate pathophysiology of SCI and advancing targeted treatments, translating these findings into clinical practice continues to pose substantial challenges (Bhatt and Das, 2025). Although methylprednisolone is the only pharmacological treatment approved by the FDA for SCI, its substantial side effects limit its therapeutic potential, emphasizing the pressing need for alternative treatment strategies (Karsy and Hawryluk, 2019). Previous studies have shown that alterations in metabolism, especially those involving glycolysis, can trigger neuroinflammatory reactions in microglia (Hua et al., 2024). This study investigated the association between GRGs and immune responses in SCI. By employing advanced analytical techniques, including gene expression analyses and machine learning algorithms, this research aimed to identify critical genes associated with glycolysis that may reflect the systemic or local inflammatory status of SCI. Key findings from this study, such as the identification of the candidate hub gene *UBTD1*, may contribute to the development of targeted therapeutic strategies that could enhance recovery following spinal injuries. Understanding the role of glycolysis in SCI provides critical insights into potential therapeutic strategies. The identification of differentially expressed DEGs such as *UBTD1 s*upports their potential significance in the inflammatory processes associated with SCI.

*IRS1* was initially identified as a candidate gene in our computational analysis. Insulin receptor substrates (IRSs) play a crucial role in mediating insulin signaling pathways, regulating key cellular processes including growth, survival, and metabolic homeostasis (Sesti et al., 2001). To date, six distinct IRSs have been identified, ranging from IRS1 to IRS6, with IRS1 and IRS2 being the most abundantly expressed in human tissues (Dearth et al., 2007). Previous investigations have demonstrated that IRS1 are significantly overexpressed in hepatocellular carcinoma and pancreatic cancer, suggesting their potential role in tumorigenesis (Nehrbass et al., 1998; Bergmann et al., 1996). Conversely, IRS1 expression has been found to be downregulated in squamous cell lung carcinoma. In contrast, IRS1 exhibits constitutive activation in various sarcomas and breast cancer, highlighting its divergent roles in different cancer types (Chang et al., 2002). Furthermore, a reduction in IRS1 expression has been observed in mouse models of Alzheimer’s disease (Fan et al., 2022). Activation of IRS1 has been shown to suppress autophagic processes and enhance locomotor recovery following spinal cord injury (Zang et al., 2023). However, in the context of SCI, the specific role of IRS1 requires further elucidation.

Ubiquitin domain-containing protein 1 (*UBTD1*) is a highly conserved protein across species, known to engage in interactions with E2 ubiquitin-conjugating enzymes within the ubiquitin-proteasome system (Liu et al., 2003; Uhler et al., 2014). In gastric cancer, UBTD1 has been shown to promote cellular senescence by enhancing the stability of the p53 protein while facilitating the degradation of Mdm2, thereby modulating key regulatory pathways involved in tumor progression (Zhang et al., 2015). Reduced expression of UBTD1 has been implicated in the progression of hepatocellular carcinoma, potentially through the modulation of the YAP signaling pathway (Yang et al., 2020). UBTD1 facilitates the progression of colorectal cancer by enhancing the stability of c-Myc, thereby promoting glycolytic activity and supporting tumor metabolism (Zhao et al., 2024). Despite the lack of studies on the role of UBTD1 in spinal cord injury, our research provides new insights into its potential involvement, suggesting that UBTD1 could emerge as a novel therapeutic target for spinal cord injury treatment. Our research provides new insights into its potential involvement, suggesting that UBTD1 could emerge as a novel candidate biomarker for spinal cord injury.

Pathway analysis further elucidates the significance of GR-DEGs in SCI. Investigating these pathways could reveal new targets for modulating neuroinflammation, which is a significant barrier to recovery in SCI patients. Moreover, the connection between glycolysis and immune regulation aligns with emerging perspectives on the metabolic reprogramming of immune cells in various conditions, including cancers and chronic inflammatory diseases (Li et al., 2025a; Li et al., 2025b; Yang et al., 2025; Deng et al., 2025). Moreover, the significant differences in immune cell populations observed using the ssGSEA method highlight the altered immune response in SCI. The increased abundance of activated dendritic cells and macrophages, coupled with a decrease in activated B cells and CD8 T cells, suggests a shift in the immune profile that may contribute to secondary injury processes. This underscores the importance of understanding how metabolic changes in glycolysis can shape immune responses and potentially inform therapeutic strategies aimed at enhancing recovery. By targeting glycolytic pathways, we may be able to restore balance within the immune landscape and improve functional outcomes in SCI patients, a notion that warrants further exploration in clinical settings.

In constructing a protein-chemical interaction network, the identification of compounds such as Testosterone and Aspirin interacting with hub genes *IRS1* and *UBTD1* offers exciting avenues for drug repurposing. Understanding these interactions could facilitate the development of novel therapeutic agents aimed at modulating glycolysis-related pathways in SCI. The implications of these findings extend beyond SCI, as they may also apply to other neurological conditions where metabolic dysregulation plays a pivotal role, highlighting the broader impact of this research on our understanding of metabolic pathways in brain injuries and diseases.

Single-cell analysis revealed that *Irs1* was primarily expressed in endothelial and epithelial cells. Additionally, we observed widespread expression of *Ubtd1* across various cell types, with particularly high levels in endothelial cells and microglia. Activation of microglia serves as a key feature of the secondary inflammatory response after SCI, with microglia-driven inflammation playing a critical role in hindering functional recovery (Devanney et al., 2020; Al Mamun et al., 2020). M1-polarized microglia, commonly induced by lipopolysaccharide (LPS), secrete pro-inflammatory factors that significantly impair neuronal viability (David and Kroner, 2011). On the other hand, M2-polarized microglia facilitate recovery by removing cellular debris via phagocytosis and releasing neurotrophic and protective molecules that promote tissue repair (Gensel et al., 2009; Lourbopoulos et al., 2015). Consequently, M2-polarized microglia enhance neuronal repair and functional recovery, while M1-polarized microglia impede these processes (Han et al., 2018). Alleviating endothelial cell senescence and enhancing recovery following spinal cord injury in diabetic mice (He et al., 2025). Acute hyperglycemia hampers the recovery of spinal cord injury by inducing excessive ferroptosis in endothelial cells (Wu et al., 2025). Following spinal cord injury, the alarmin interleukin-1α initiates secondary degeneration by activating reactive astrocytes and endothelial cells (Bretheau et al., 2022). Collectively, these findings highlight the intricate interplay between endothelial cells, epithelial cells, microglia, and inflammatory mediators in the pathophysiology of spinal cord injury. This understanding may open new avenues for the development of therapeutic strategies targeting spinal cord injury. These findings highlight the potential roles of *IRS1* and *UBTD1* in SCI pathophysiology. However, while the dataset includes valuable temporal data, this study focused on static expression patterns at a single time point, with the goal of providing insights into gene expression at the cellular level. To gain a deeper understanding of the dynamic processes involved in SCI, future studies should leverage the temporal structure of this dataset to investigate dynamic gene regulation, cell-state transitions, such as microglial polarization, and cell–cell communication mechanisms. These analyses will be essential for uncovering the underlying mechanistic processes that drive SCI progression and recovery.

To experimentally validate the computational findings, we performed qRT-qPCR and Western blot analyses to compare the expression levels of IRS1 and UBTD1 in spinal tissues from the SCI and Sham groups. *Ubtd1* expression was significantly elevated in the SCI group relative to the Sham group, consistent with the bioinformatic predictions. In contrast, *Irs1* showed no significant alteration in the injured spinal cord tissue. This discrepancy between the blood-based computational prediction and the tissue-based experimental validation suggests that *IRS1* dysregulation might be context-dependent. It is possible that *IRS1* alterations are more prominent in the systemic immune circulation (as detected in the discovery dataset) than in the local spinal cord microenvironment, or that its cell-type-specific expression (in endothelial cells) was masked by the complex cellular heterogeneity of bulk tissue samples. Consequently, given the robust consistency of *UBTD1* across all analytical layers, we prioritize *UBTD1* as the primary candidate hub gene for future investigation.

This study acknowledges several limitations that may impact the generalizability and robustness of the findings. The relatively small sample size may limit the statistical power and the ability to detect subtle but clinically significant effects. Furthermore, the lack of clinical validation analysis raises concerns regarding the translational potential of the identified biomarkers and therapeutic targets.

In addition, the discovery and validation datasets differed in both tissue origin and species, with peripheral blood transcriptomes from human subjects and spinal cord tissues from a rat model, respectively. Peripheral blood gene expression primarily reflects systemic immune and inflammatory states, whereas spinal cord tissue represents the local microenvironment of the central nervous system. Therefore, the validation analyses were restricted to transcriptomic and pathway-level consistency rather than tissue-specific or mechanistic confirmation, and the findings should be interpreted with caution in this context.

Lastly, potential batch effects resulting from the integration of multiple gene expression datasets could introduce confounding variables, necessitating cautious interpretation of the results. Moreover, the current study did not include *in vitro* cellular experiments to validate the functional roles of the identified genes. While these genes were identified through computational analyses, the lack of experimental validation in cell-based models is a limitation, and future studies should include these to confirm the mechanistic roles of the identified key genes in spinal cord injury.

Collectively, these limitations underscore the need for further studies incorporating larger cohorts matched human spinal cord tissues, longitudinal clinical validation, and *in vitro* cellular experiments to further corroborate the current findings and refine our understanding of metabolic–immune interactions in spinal cord injury.

## 5 Conclusion

In conclusion, this study systematically investigated the association between glycolysis-related genes and immune responses mechanisms in spinal cord injury through integrative transcriptomic and experimental analyses, providing pathway-level insights into metabolic alterations associated with SCI. The identification of the hub gene UBTD1 highlights its potential value as candidate biomarker, rather than a definitive mechanistic driver, and points to avenues for future research and hypothesis-driven intervention strategies.

By leveraging advanced analytical techniques, this study adds to the growing body of evidence linking metabolic reprogramming with immune dysregulation in SCI, and suggests that glycolysis-related pathways may represent important components of the post-injury inflammatory landscape. Continued exploration of these findings in larger cohorts, using matched human spinal cord tissues and additional functional experiments, will be essential for translating these observations into clinical practice and ultimately enhancing outcomes for individuals affected by SCI.

## Data Availability

The original contributions presented in the study are publicly available. These data can be found in the NCBI Gene Expression Omnibus (GEO) with the accession numbers GSE151371, GSE45006, and GSE162610. Further inquiries can be directed to the corresponding author.
